# Investigation into the Three-Stage Formation of Micro-Channels with Ultra-Thin Titanium Sheets Used for Proton-Exchange Membrane Fuel Cell Bipolar Plates

**DOI:** 10.3390/ma17051071

**Published:** 2024-02-26

**Authors:** Youfu Xie, Xiao Fang, Chunju Wang, Qi Zhong, Yucheng Wang, Risheng Hua

**Affiliations:** 1 Bosch Automotive Systems Co., Ltd., Wuxi 214028, China; 2 Bosch Powertrain Systems Co., Ltd., Wuxi 214028, China; 3 Robotics and Microsystems Center, School of Mechanical and Electric Engineering, Soochow University, Suzhou 215131, China

**Keywords:** fuel cell, bipolar plate, titanium, three-stage forming

## Abstract

Titanium has a low density and high corrosion resistance. In order to achieve the goal of a lightweight material, and to extend the normal working hour of proton-exchange membrane fuel cells (PEMFCs), ultra-thin titanium plates were chosen to manufacture the key components—bipolar plates (BPs). For the purpose of overcoming the challenges of manufacturing with a large depth to width ratio, a multi-stage formation process was established with characteristics such as high efficiency and a lower price. In this study, the process parameters were examined through an experimental approach. The outcomes show that the channel formed by multistage forming is deeper than that formed by single-stage forming under the same displacement conditions. To achieve greater flow depths, it is recommended to increase the displacements as much as possible during both the first- and second-stage forming processes. The implementation of three-stage forming can effectively reduce the maximum thinning rates within flow channels while improving the overall deformation uniformity. This method deviates from traditional one-stage loading processes by adopting multi-stage loading instead. By employing appropriate mold designs, material deformation and flow can be enhanced throughout gradual loading processes, thereby preventing strain concentration and enhancing the ultimate formation height accuracy within micro-flow channels. Consequently, the proposed three-stage forming process proves highly appropriate for the mass production of BPs utilizing titanium plates.

## 1. Introduction

In order to build an environmentally friendly social environment and reduce the consumption of fossil energy, new types of renewable green energy have been widely studied. One such energy source is hydrogen, which is regarded as a promising solution for energy in a few years. It is estimated that when hydrogen is used, greenhouse gas emissions will be reduced to near zero if it is treated as a renewable energy source [1].

Usually, the most critical components of a fuel cell stack include the following two parts: BPs and the membrane electrode assembly (MEA) [1]. The BPs account for 30–50% of the cost of the whole stack, 60–90% of the volume, and 60–85% of the weight [2]. The functions of the BPs are as follows: conveying the reaction gas, transmitting a current, transferring waste heat, cooling water management, etc. [3]. Conventional BPs are manufactured by graphite machining. However, because graphite is too brittle, it cannot adapt to the manufacturing methods and working conditions it needs to withstand when processing and working. Additionally, the price of graphite is not cheap, the material utilization rate is low in the manufacturing process, and there are high amounts of waste. Recently, the market share of metal BPs has been gradually increasing. Conventional metal bipolar plate processing methods include stamping, thermoforming, hydroforming, powder metallurgy molding, electromagnet forming, ultrasonic assisted forming, etc. [2]. The metal BPs processed by the stamping process have a low cost, high strength, and high production feasibility, and have been applied to automobiles with high power density requirements, such as Toyota Mirai and Hyundai NEXO, which have been sold in the market. In the processing of metal BPs, materials with good corrosion resistance, good conductivity, and high strength, which are easily processed, are expected to be used as the raw material of BPs [4]. Karacan, Liu, and Wilberforce et al. investigated the feasibility of processing metal BPs from 316 stainless steel, 304 stainless steel, Al6061 aluminum alloy, Copper, and TA1 pure titanium. According to their analysis, titanium alloys are an ideal candidate. They have many characteristics required for BPs, such as high electrical conductivity, specific strength, and corrosion resistance [5,6,7]. Fiedler et al. conducted a study on the stability of titanium and 316 L stainless steel BPs under simulated conditions of deionized water and 0.5 mM H_2_SO_4_ [8]. Celik et al.’s research compared formed stainless steel BPs, additively manufactured titanium BPs, and machined graphite BPs with identical flow channel shapes. Additionally, gold-plated titanium BPs were examined and their properties were compared as well. Among the unit cell tested, the 450 nm gold-plated titanium BP cell had the highest peak power of 639 mW/cm^2^ [9]. In the field of membrane electrode research, A. Villagra et al. studied PEM (proton exchange membranes) hydroelectrolysis batteries at high current density. Under the condition of a working temperature of 80 °C, the polarization curve is tested with a different thickness of PFSA (perfluoro-sulfonic acid), and the current density reached 10 A/cm^2^ [10]. Graphene is believed to enhance the conductivity of PEM, and D. Ion-Ebrasu et al. investigated the water absorption and ion exchange capacity of membranes with different graphene contents. Under certain conditions, a membrane loaded with 10 mg of graphene can achieve an activation energy of 3.8 kJ/mol for proton transfer [11].

Many scholars have conducted research on the processing methods of metallic BPs. Talebi-Ghadikolaee et al. conducted a study on the forming process of 316 stainless steel metal BPs and simulated it using finite element software. The simulation results were validated by comparing the thickness distribution and force–displacement curve. Experimental observations revealed that rupture occurred at a channel depth of 0.610 mm. The critical limits for the equivalent strain and thinning rate were determined to be 0.56 and 33.45%, respectively, which can serve as acceptable ranges for plastic deformation during conventional stamping processes of BPs [12]. Minh et al. explored the two-stage forming process of pre-forming and final forming to enhance the formability of metal BPs. The finite element simulation methods of microforming and large forming were established, and the influence of stamping die on the formability of ultra-thin metal BPs was studied. Finally, a new mold was designed, which can prevent the partial cracking of parts and distribute the thickness evenly [13]. Xu et al. researched the three-stage forming of channels of BPs, pointed out that the titanium plate has strong anisotropy, and revealed how the multi-stage forming process can improve the ultimate forming depth of the micro-channels. The multi-stage forming process can obtain a higher drawing depth, and the maximum length-diameter ratio can reach 0.67. In addition, the experiment shows that the interface contact resistance of the BPs prepared by single-stage forming is higher than that of the BPs prepared by multi-stage forming. Compared with the single-stage BPs, the interface contact resistance of the three-stage BPs decreases from about 33 mΩ·cm^2^ to about 24 mΩ·cm^2^ at 0.2 MPa pressure [14]. Through experiments and simulations, the multi-stage forming of micro-channels with a thin width and high drawing ratio was realized by Zhang. Three key parameters of the stamping process are put forward: stamping displacement, die fillet size, and die clearance. The quality of the forming process can be quantitatively analyzed using several quality evaluation indexes, such as the height of the channel, the thickness of the sheet, and the angle of drawing. The experimental results show the advantages of multistage forming in improving various indexes. The thickness of the sheets in the three-stage forming are more uniform. The minimum thickness increased from 0.065 mm to 0.071 mm [15]. Mohammad et al. experimentally studied the influence of the stamping force on the dimensional precision and thickness uniformity of the channel of the stamped BPs. It was found that for a bipolar plate with an active area of 100 cm^2^, when the compression force increases from 100 tons to 160 tons, the rib width increases from 0.97 mm to 1.1 mm. The increase in the clamping force can increase the channel depth and rib width [16]. Bong et al. used two different sheets with thicknesses of 0.075 and 0.1 mm for two-stage forming experiments and finite element modeling to vindicate the improved formability of the two-stage forming. The finite element calculation results show that the formability of the micro-channel is improved with the increase in the punching radius in the first stage [17]. Talebi Ghadikolaee et al. investigated the stamping process of metal BPs under lubricated and non-lubricated conditions. Their findings indicated that in the finite element analysis, accurate thickness prediction results could be obtained on lubrication conditions with a friction coefficient of 0.1 [18].

Zhong et al. researched the influence of the die fillet size, slot width to rib width ratio, and stamping depth on BPs multi-stage forming through finite element simulation and experiments. The punch is optimized to be an arc, which can decrease the thinning rate from 47 to 22%. Considering only the thinning rate, the best ratio between rib and channel width was 0.4–0.6 [19]. Qiu et al. established a new approach for evaluating the formability and reaction efficiency of BPs, called the response surface method (RSM). Based on this approach, a rib width of 0.9 mm, flow channel width of 0.9 mm, radius of 0.15 mm, and channel depth of 0.4 mm are proposed when the thickness of the sheet metal is 0.1 mm [20]. Khatir et al. studied the influences of the external dimension of BPs on the formation of sheets. The effects of various parameters on the substrate thickness and channel depth of BPs were evaluated through the experiments. The research data show that the parameters that have the greatest influence on the thinning rate are channel depth, fillet size, and draft angle. The contribution percentages were determined to be equal to 38.1, 28.9, and 19.6%, respectively [21]. Modanloo et al. tried to select the optimal forming conditions for the manufacture metallic BPs from experiments with different criteria. By assimilating process parameters such as the mold matching gap, drawing speed, and lubrication, 15 experiments were designed as alternatives. Through the TOPSIS and VIKOR analysis, it was pointed out that the optimum forming conditions for the titanium BPs are as follows: gap is 0.2 mm, speed is 3.5 mm/s, and friction coefficient is 0.2 [22]. Karacan et al. studied the formation and simulation of four different metal materials—Al 3104, Al 6016, CP-Ti, and SS 304—and four different flow field designs. They concluded that CP-Ti and SS 304 are suitable for 0.36 mm flow channel depths, while CP-Ti materials cannot be candidates for deeper flow channel applications [5]. Modanloo et al. prepared parallel flow field titanium BPs using the stamping process, and studied fracture prediction in stamping processes using ductile fracture criteria. The experimental data show that the Brozzo ductile fracture criterion is the most reliable standard to evaluate the formation quality of titanium BPs, and the accuracy is 96.32% compared with the experiment [23]. Park et al. optimized the formability of BPs by adjusting conditions such as the sheet type (SUS 304 and SUS 316, force load conditions, and heat treatment. The experimental data show that when the maximum static load and the maximum dynamic load are both 220 kN, the channel depth of the dynamic load increases by 22 μm. Compared with the non-heat-treated plate, the channel of the heat-treated plate on BPs is deeper and the wall thickness is more uniform. Compared with SUS 316, the formability of the SUS 304 material is better [24].

Furthermore, there are different processing techniques that can produce metal BPs, for example, hot-stamping process technology [25,26], ultrasonic-assisted vibration stamping technology [27], the rubber pad forming process [28], hollow embossing rolling [29], and hydraulic forming. Modanloo et al. experimentally studied the influence of forming temperature and velocity on the formability of hot-stamping BP titanium alloy. The results show that the formability of sheet metal is related to the deformation temperature and deformation speed. When the temperature of sheet metal and die is raised from room temperature to 100 °C, the rebound rate of ultra-thin titanium sheets can be reduced and the drawing depth of the formed sheet can be increased. At 100 °C, the maximum channel depth of the titanium BPs is 0.494 mm. At room temperature, the maximum channel depth is 0.373 mm [25]. Guo et al. determined the effects of the mold temperature, friction coefficient, and raw material grain grade on BP formation. Hot-stamping improves the size precision and forming stability of ultra-thin SS316L BPs. The optimum process temperature for a grain size of 10.7 μm is 1000 °C. Compared with room temperature stamping, the dimensional deviations of the ultra-thin SS316L bpp of channel height, rib width, and fillet size are reduced by 8.6 μm, 19.8 μm, and 28.3 μm, respectively [26]. Wang et al. performed an ultrasonic-vibration-assisted stamping of BPs. When the ultrasonic power is 70%, the microchannel depth is about 100 μm larger than that without ultrasonic vibration. With the increase in the duration, the microchannel depth becomes larger. The duration of the “Blaha effect” is 1.5 s, and the depth of the microchannel is increased by about 62 μm compared to that without ultrasonic vibration [27]. Talebi-Ghadikolaee et al. investigated the forming method of the rubber pad forming process for ultra-thin BPs made of SS316L stainless steel with a thickness of 0.1 mm, determining the appropriate channel depth and studying the molding process function of rubber pads [28]. Reuther et al. suggested that hollow embossing rolling is a promising metal BP forming technology and its finite element simulation method was studied. It was found that the representation of manufacturing-related changes in rolling clearance is crucial to the accuracy of the numerical model [29]. Holger J. et al. demonstrated a simple and cost-effective fabrication method for titanium BPs which reduced contact resistance by approximately 75%. The contact pressure distribution on the active area was tested and found to be uniform (±1.25 MPa) [30].

In our study, the titanium metallic BPs undergoing a three-stage formation process were studied using an experimental method. In a previous study [19,27], a three-stage forming mold was designed. Firstly, the influence of the clamping force on the shape of the three-stage-forming flow path was studied. Then, the law of the mutual influence between the forming states of each stage in the three-stage forming was studied. The research results provide a significant reference for the development of a multi-stage forming process of titanium BPs for fuel cells.

## 2. Experimental Results and Analysis

### 2.1. Material Parameter

**Figure 1:**
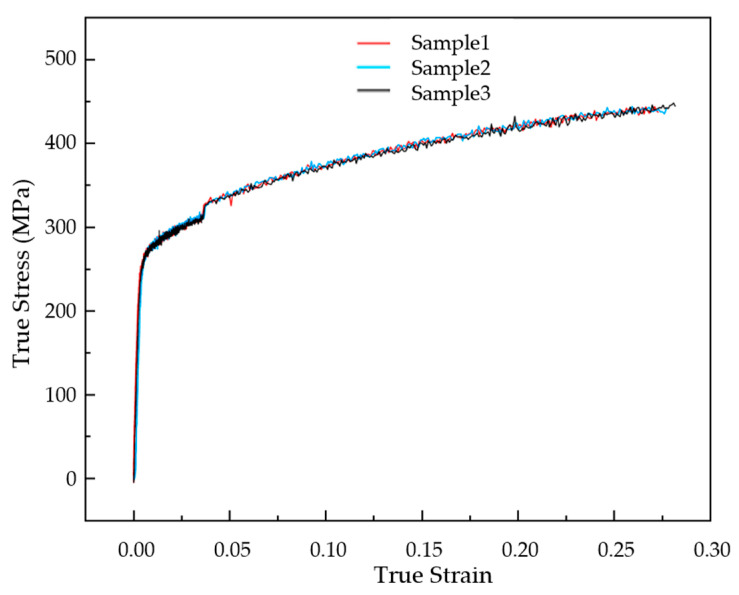
Curves of true strain–stress of TA1.

Titanium is a kind of metal material, with a hard material, light weight, corrosion resistance, and other characteristics. It is widely used in aviation, aerospace, navigation, and other industries. Because of the above characteristics, it is also suitable for the processing of metal BPs, which can help to achieve lightweight vehicles and improve the power density of fuel cells. Our team has conducted some studies on titanium plates at the early stage [19,27]. In this study, the same 0.1 mm thickness titanium material is used as in the previous research. Its material compositions are shown in Table 1, and its general material properties are shown in Table 2 [19]. The test sample was prepared with reference to the standard ASTM E8/E8M-11 [31], and a uniaxial tensile test was carried out with AGS-X10KN equipment (Shimadzu, Kyoto, Japan). The gauge length of the test sample is 32 mm and the width is 6 mm. The stress–strain curve results are shown in Figure 1.

### 2.2. Experimental Device and Mold Design

**Figure 2:**
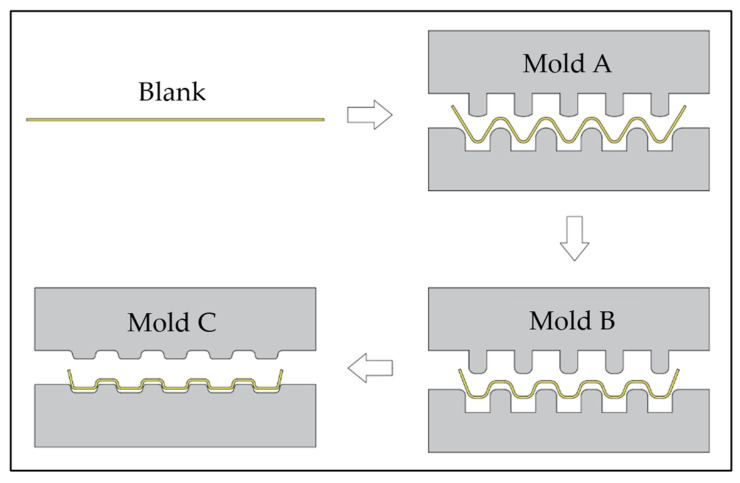
Multi-stage forming process.

To explore the multi-stage forming process of titanium BPs, a multi-stage experiment was established in this study; the experimental process is shown in Figure 2. The same sample was pressed successively according to the fillet radius, moving from large to small, using mold A, mold B, and mold C, respectively. The findings of the study show that the multi-stage forming is superior to the results of single-stage forming using mold C to evaluate the benefits of multi-stage forming in terms of the high depth to width ratio, fine flow channel structure, and uniform channel wall thickness.

**Figure 3:**
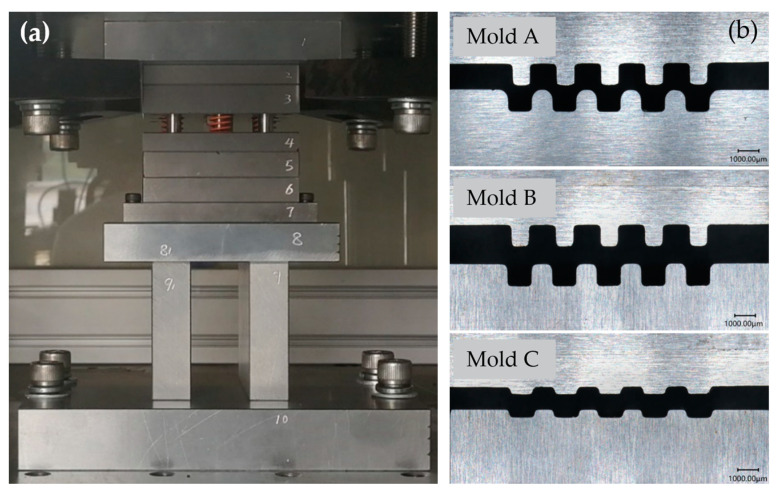
Mold for multi-stage formation of BP channels. (**a**) Picture of stamping mold, (**b**) punch and die.

**Figure 4:**
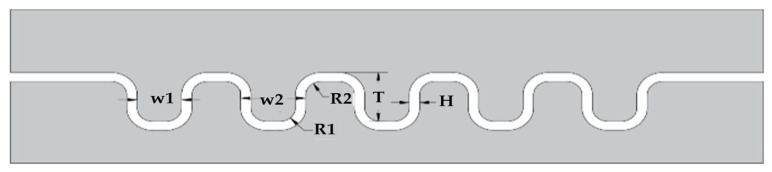
Schematic diagram of channel size annotation.

The mold used in this experiment was a three-stage forming mold, as shown in Figure 3. The sizes of the stamping molds are shown in Figure 4 and Table 3 [19].

A self-developed, servo-motor-driven press was used in the experiment, and the displacement of the press was measured with a grating ruler. Confocal microscopy (VHX-7000, Keyence, Osaka, Janpan) was used to analyze the size and cross-section microstructure of the formed microchannels. To ensure the samples had the same flatness and consistency, the samples were prepared by wire-cutting.

### 2.3. Experimental Process and Results

Before the experiment, it is necessary to ensure that the mold has a uniform displacement starting point. First, place a sample in the mold and control the punchdown after adjusting the displacement value to zero. When the pressure value reaches 10 N, it should be stopped immediately. This position is the point at which the default punch and plate begin to make contact. This position is the fixed starting point of the first forming stage and the second forming stage. However, it should be noted that, when setting the amount of pressure, it is necessary to add 0.5 mm that is reserved for the operating space. The third stage of forming, as the last stage, directly processes the shape of the flow channel and the flow channel shape, and the third stage of the forming process is controlled by pressure. After the experiment, the molds are returned to the starting position.

**Figure 5:**
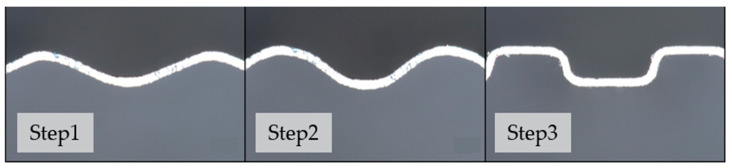
Flow channel cross-section of the forming process.

The control of the mold parameters during the experiment is shown in Table 4. In the first two stages, it is necessary to ensure that each experiment has the same pressing distance and stamping speed. Each group of experiments is repeated 10 times. The pressure value of the third stage needs to be studied to ensure the dimensional accuracy of the channel. The actual stamping process is shown in Figure 5.

**Figure 6:**
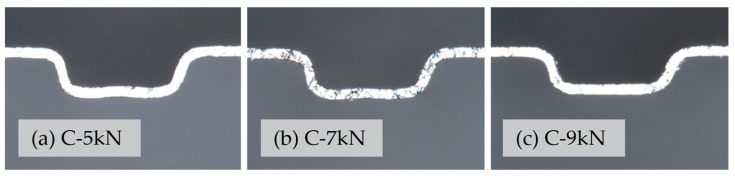
Flow channel cross-section of the third stage of the forming (using the C mold) under different closing forces.

First, the clamping force of the third stage is studied using the sample (group 21) with the largest displacement of the first and second stage punches. The clamping force of the third stage is controlled to 5 KN, 7 KN, and 9 KN, respectively. The test data of the forming samples are shown in Figure 6.

It is easy to see from Figure 6 that, if the deformation of the first two stages is large and the pressing pressure in the third stage is not enough, a blank will appear, as shown in Figure 6a, giving the bottom of the groove a “low-high-low” shape. This phenomenon is mainly because the first two stages exceed the depth of the C-mode, the pressure in the third stage is not enough, and the punch cannot flatten the excessive deformation. With the increase in the clamping force, the bottom of the flow channel tends to gradually become flat, as shown in Figure 6c. When the pressure value reaches 9 KN, the bottom of the groove is relatively flat, and this phenomenon has basically disappeared. Therefore, 9 KN was selected as the third-stage press pressure.

#### 2.3.1. The Effect of Process Parameters on the First Stage

**Figure 7:**
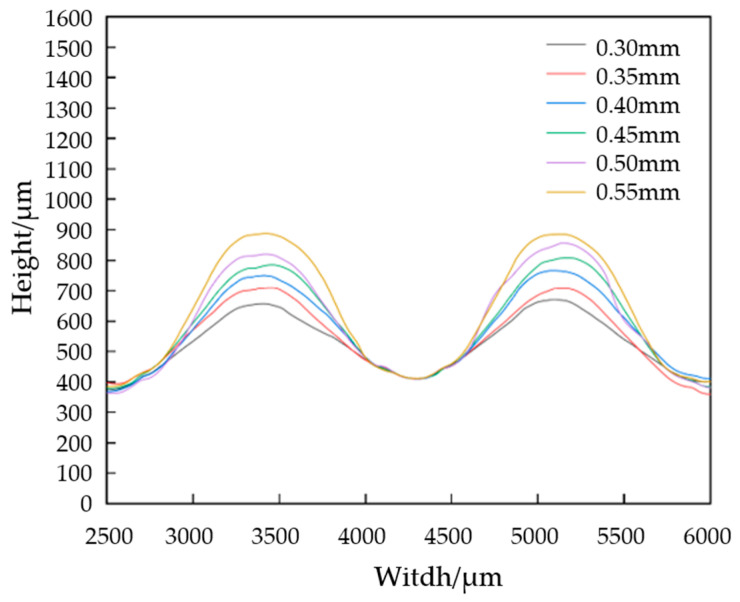
First-stage forming channel profile for different press displacements.

**Figure 8:**
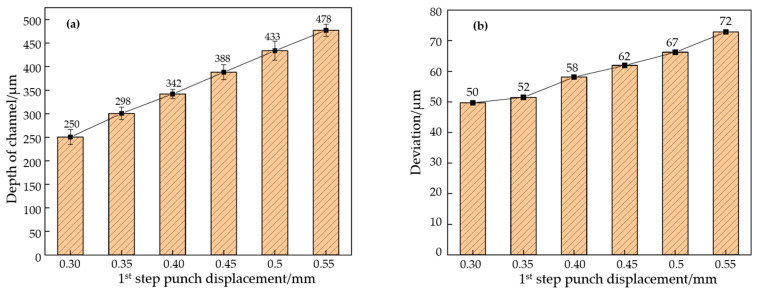
The channel size after first formation. (**a**) The channel depth for different punch displacements, (**b**) the depth deviation for different punch displacements.

After each stamping step is completed, the sample is scanned with an ultra-deep microscope to measure and sort the outline dimensions. The sample size after the first stage of stamping is shown in Figure 7. It can be seen from the contour curve that the flow channel after the first stamping stage presents a “peak and groove” shape without platform structure characteristics, and the flow channel depth gradually increases with the increase in mold displacement. By measuring the depth of each channel and calculating its average value, the accurate depth of the channel is obtained. The average values are shown in Figure 8a, and the springback amounts are calculated, combined with the punch displacement. The results are shown in Figure 8b. With the increase in upper mold displacement, the increase in flow channel depth is nearly linear. When the punch displacement is 0.30 mm, the height of the channel is 0.25 mm, and the deviation is 50 μm. When the punch displacement is 0.55 mm, the height of the channel is 0.478 mm, and the deviation increases to 72 μm. Springback is one of the reasons for the deviation of the flow channel. The amount of springback will increase with the increase in the depth of the flow channel. This is because the greater the deformation of the plate, the greater the strain gradient of the rounded corner area that is generated, so the springback value will increase when the load is released.

#### 2.3.2. The Effect of Process Parameters on the Second Stage

**Figure 9:**
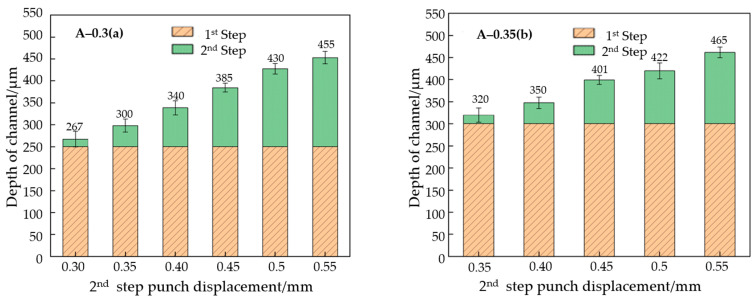

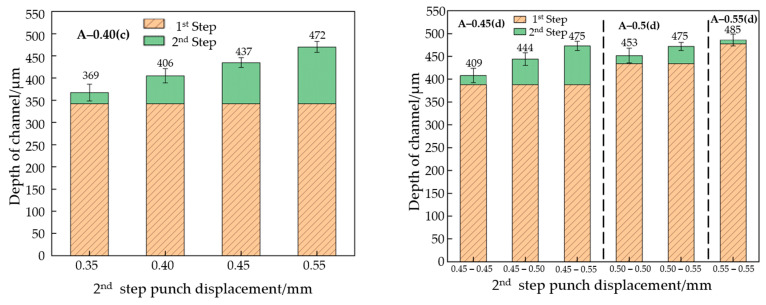
The increase value of the flow channel depth after the second stage of forming. (**a**) A mold displacement of 0.30 mm, (**b**) a mold displacement of 0.35 mm, (**c**) a mold displacement of 0.40 mm, (**d**) mold displacements of 0.45 mm, 0.50 mm, and 0.55 mm.

In Figure 9, the height of the channel results for the second stage is shown. After the second-stage stamping, the depth of the flow channel increased on the basis of the first stage. Even in the case where the first and second stages have the same stamping distance, the flow channel depth of the second stage is still 10–20 μm higher than that in the first stage. After the end of the second stage, the shallowest flow channel is found in the A-0.30 mm and B-0.30 mm group, where the depth is 267 μm, and the deepest flow channel is found in the A-0.55 mm and B-0.55 mm group, where the depth is 485 μm.

**Figure 10:**
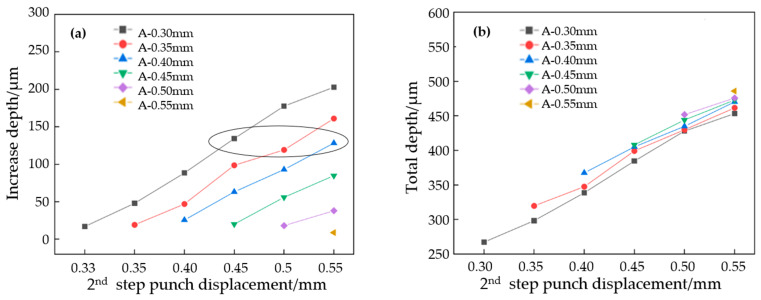
Depth of the flow channel after second-stage forming. (**a**) Flow channel’s added depth, (**b**) total depth of channel.

In Figure 10a, the depth increases in the flow channel of the first-stage forming, with different depths than those found under the different punch displacement of the second stage. It can be seen that, under the same A-mold displacement, the depth of the channel addition increases nearly linearly with the increase in the upper mold displacement of the second stage. Transverse comparison shows that, during the second stage of displacement, the smaller the pressure in the first stage, the greater the increase in the height of the channel. The deformation of the first stage needs to be taken into account. The three pieces of data in the third row of Figure 10a are all pressed 0.15 mm again upon the original deformation, and the increase in the depth of the flow channel shows no obvious rule. This is mainly because the increase in depth is too small, and the difference in springback is not obvious.

Figure 10b shows the relationship between the total height of the channel and the upper mold displacement after a two-stage forming process. It can be seen that the final depth of the experimental group of mold A 0.30 mm and mold B 0.55 mm is lower than that of the experimental group of mold A 0.55 mm and mold B 0.55 mm. In the second stage with the same punch displacement, the deeper the forming depth of the first stage, the deeper the final forming depth of the flow channel. In other words, the greater the deformation in the first stage, the smaller the springback in the end. Therefore, in order to improve the depth of the flow channel, the depth of the first stage should be increased as much as possible.

**Figure 11:**
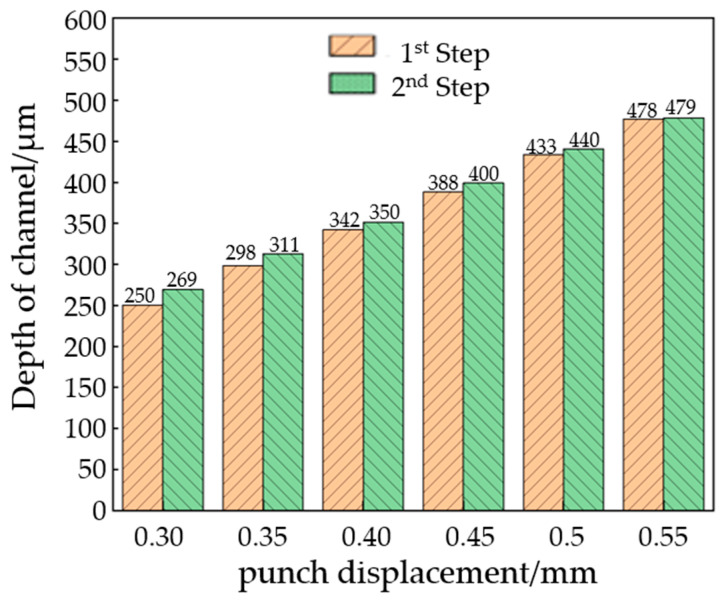
Flow channel depth comparison between stage 1 and stage 2.

The average value of the flow channel depth with the same second-stage punch displacement is calculated and compared with that of the first stage. The results are shown in Figure 11. Under the 0.30 mm punch displacement, the channel depth that occurs with the single-stage forming is significantly lower than that which occurs with the two-stage forming, while under the 0.55 mm punch displacement, the channel depth of the single-stage and the two-stage formations is almost the same.

Although the overall two-stage forming depth is higher than that of the single-stage forming, the gap between the two stamping methods gradually shrinks as the displacement of the punch increases. When the upper mold displacement is small, the two-stage forming can significantly improve the channel depth, but when the punch displacement is larger, the improvement effect is not obvious. This is mainly because when the material tensile reaches beyond a certain range, the forming quality is no longer determined by the forming process, but by the performance of the material itself.

#### 2.3.3. The Effect of Process Parameters on the Third Stage

**Figure 12:**
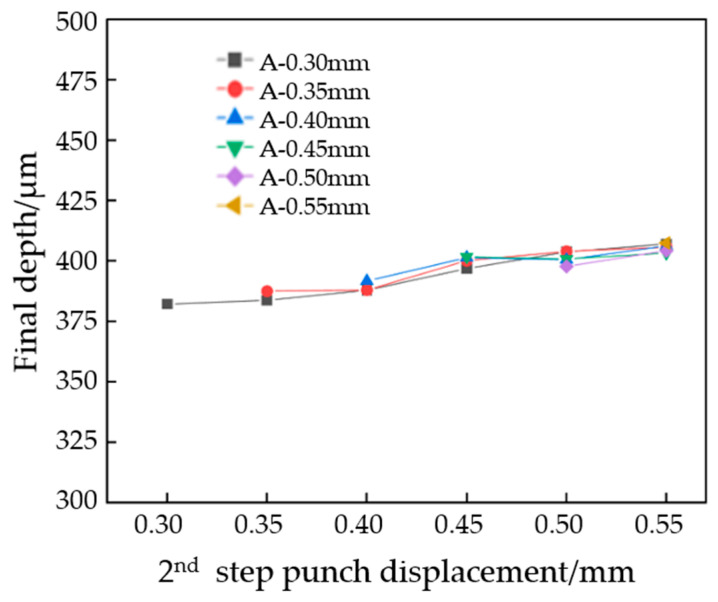
Depth of flow channel for different punch displacements after two-stage forming.

**Figure 13:**
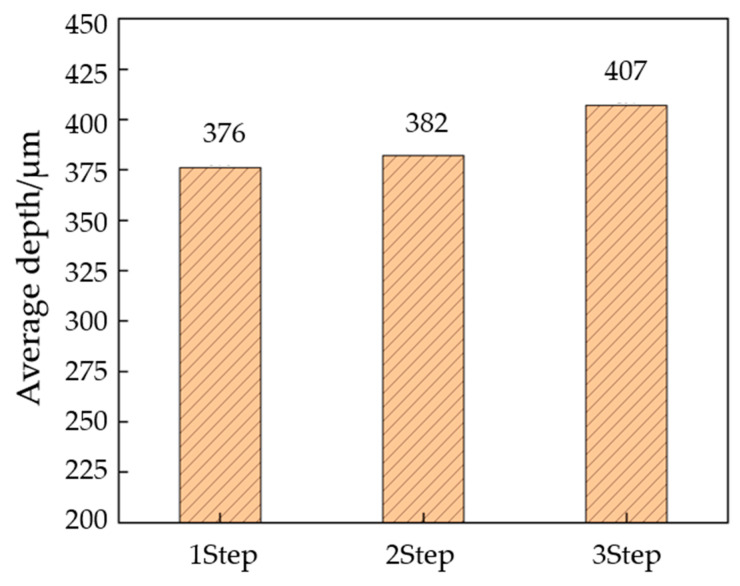
Flow channel depth comparison between stage 1, stage 2, and stage 3.

The samples that were formed in two stages are pressed in the third stage. In the first two steps, the clamping force was set to 9000 N and held for 180 s. The measurement results are shown in Figure 12. Overall, with the increase in pressure in the first two stages, the flow channel depth also increases. Most of the experimental channels reach the designed depth of 400 μm. The shallowest flow channel is found in the group with A mold 0.30 mm and B mold 0.30 mm, where the depth is 382 μm, and the deepest flow channel is found in the group with A mold 0.55 mm and B mold 0.55 mm, where the depth is 407 μm. Therefore, in order to obtain the maximum channel depth, the punch displacement of the first and second stages should be increased as much as possible. Figure 13 compares the depth of the stamping parts after single-, two-, and three-stage forming. It can be seen that the effect of the three-stage forming process on the forming depth is universal, and the minimum can be increased by 6 μm and the maximum can be increased by 31 μm compared with the single-step forming.

Because of the small value of the drawing ratio in the mold design, the channel can be completely formed without breaking when using a multi-stage forming process, so that the single-stage forming can be stamped close to the design depth. When summarizing the best process parameters of the three-stage forming process, it is impossible to accurately analyze the pros and cons of the multi-stage forming through comparing the forming depth. Therefore, in this study, the three-stage forming quality analysis is evaluated by comparing the thinning rate of the blank at the same punching distance or pressure value.

**Figure 14:**
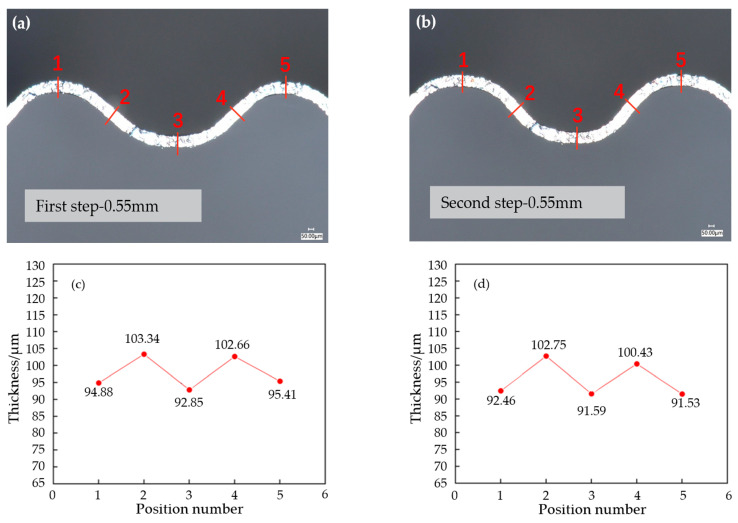
Blank thickness after stage 1 forming and stage 2 forming. (**a**) Blank thickness after stage 1, (**b**) blank thickness after stage 2, (**c**) blank thickness value after stage 1, (**d**) blank thickness value after stage 2. (Note: The number 1–5 in figure (**a**,**b**) corresponds to position number in figure (**c**,**d**)).

To study the effect of a three-stage forming process on blank thickness, samples with a punch displacement of 0.55 mm in the first and second stages were selected. The section photos of the samples after each stage were taken using the ultra-depth field microscope, and the thickness was measured. The results are shown in Figure 14. After the end of the first stage, both the channel ridge and groove showed thinning, and the thinning rate was about 5–8%.

The maximum strain occurred in the rounded corner area, and the maximum thinning was found in the middle of the channel, while the lateral wall thickness of the channel did not change significantly. After the end of the second stage, the thinning rate of the runner increased compared to that of the first stage. The rounded corner area still underwent the most thinning, and the maximum thinning rate reached 8.5% at the middle ridge of the right runner.

**Figure 15:**
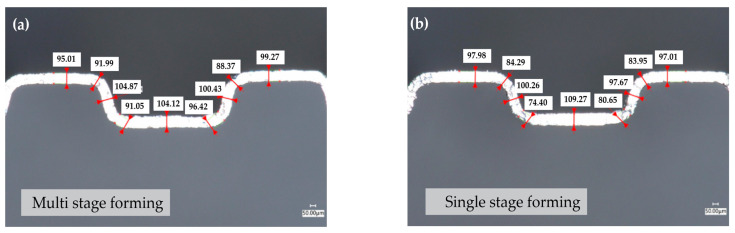
Blank thickness after single-stage forming and multi-stage forming. (**a**) Single-stage forming, (**b**) multi-stage forming.

After the third-stage forming, the flow channel was completely formed, as shown in Figure 15a. The material was mainly thinned at the rounded corners, and the maximum thinning amount was 12% of the upper-right rounded-corner area of the flow channel. It can be seen that the increase in the three-stage-forming fillet thinning rate is relatively gentle, and the increase in each stage is about 4%. The wall thickness of the single-stage forming specimen was measured. As shown in Figure 15b, it can be found that, in the single-stage forming, the thinning strongly changed, and the maximum thinning rate reached 26%, which fully indicates that the three-stage forming effectively reduced the maximum thinning rate of the channel and improved the channel-forming ability.

**Figure 16:**
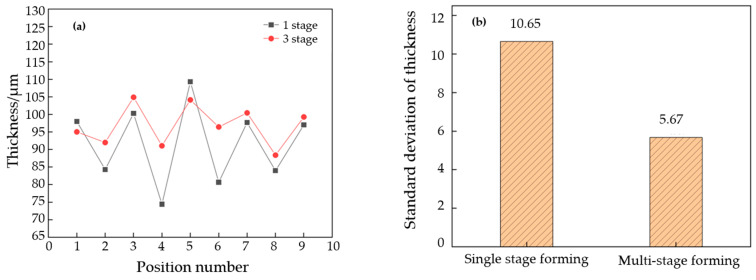
Thickness data statistics for single-stage forming and multi-stage forming. (**a**) Uniformity comparison, (**b**) standard deviation.

In order to study the difference in the uniformity of the thickness distribution between three-stage forming and single-stage forming, in Figure 16, the thickness distribution is plotted as a corresponding scatter plot, and the standard deviation of the thickness data was evaluated to quantify the uniformity. As shown in Figure 16a, the variation range for thickness in the three-stage forming is lower than that in single-stage forming. Except for positions No. 1 and No. 5, the thinning rate in the three-stage forming is lower than that in the single-stage forming. The strain concentration is obvious in the two lower fillet corner regions (No. 4 and No. 6 positions) of the single- stage forming, and the blank thickness is too low compared with other positions, resulting in poor overall wall thickness uniformity. In Figure 16b, the standard deviation of the thickness distribution in the three-stage forming is 5.67 μm, while that in the single-stage forming is 10.65 μm. The results show that a three-stage forming can improve the overall deformation uniformity, and thereby increase the overall forming depth.

## 3. Summary

In this work, the processing of titanium fuel cell BPs was studied, and several parameters of the multi-step forming process, such as punch displacement and clamping force, were discussed. A BP channel structure with a more uniform thickness and deeper depth was obtained. The conclusions are as follows:

(1) When the first stage is formed, the springback amount of the channel after forming is a linear relationship to the punch displacement of the mold. When the punch displacement is 0.3~0.55 mm, the springback amount of the flow channel is 13~20%.
(2) In the process of multi-stage BP machining, the increase in punch displacement in the first stage can reduce the rebound of subsequent processes by 10%.
(3) The standard deviation of material thickness after single-stage forming is 10.65 μm. The uniformity of the thickness is doubled after optimizing this for three-stage forming. And the standard deviation is reduced to 5.67 μm. The outcomes indicated that the three-stage forming can enhance the overall deformation uniformity of the billet and increase the overall forming depth.

## Figures and Tables

**Table 1:** Chemical compositions of TA1.

Element	Ti	Si	Fe	C	N	H	O	Others
Weight%	≥99.20	≤0.10	≤0.15	≤0.05	≤0.03	≤0.015	≤0.15	≤0.30

**Table 2:** Basic material properties of TA1.

Density	Young’s Modulus	Poisson’s Ratio
4.51 g cm^−3^	80 GPa	0.38

**Table 3:** Dimensions of dies (mm).

NO.	T	H	*w*_1_	*w*_2_	R1	R2
A	1.00	0.165	0.82	1.15	0.85	0.40
B	1.00	0.165	0.82	1.15	0.30	0.30
C	0.40	0.08	1.00	1.20	0.15	0.15

**Table 4:** Three-stage forming process route.

NO.	1st Stage Punch disp./mm	2nd Stage Punch disp./mm	3rd Stage Closing Force/N
1	0.30	0.30	5000 7000 9000
2	0.30	0.35	
3	0.30	0.40	
4	0.30	0.45	
5	0.30	0.50	
6	0.30	0.55	
7	0.35	0.35	
8	0.35	0.40	
9	0.35	0.45	
10	0.35	0.50	
11	0.35	0.55	
12	0.40	0.40	
13	0.40	0.45	
14	0.40	0.50	
15	0.40	0.55	
16	0.45	0.45	
17	0.45	0.50	
18	0.45	0.55	
19	0.50	0.50	
20	0.50	0.55	
21	0.55	0.55	

## Data Availability

Data are contained within the article.
